# Preventive behaviors of COVID-19 in the Iranian adult population during the pandemic: Integrated effect of the health belief model and the planned behavior intention using causal path analysis

**DOI:** 10.22088/cjim.15.1.13

**Published:** 2024

**Authors:** Erfaneh Hajian-Tilaki, Karimollah Hajian-Tilaki, Afsaneh Bakhtiari

**Affiliations:** 1 Student Research Committee, Roozbeh Psychiatric Hospital, Tehran University of Medical Sciences, Tehran, Iran; 2 Department of Biostatistics and Epidemiology, School of Public Health, Babol University of Medical Sciences, Babol, Iran.; 3 Health Center of Social Determinants, Health Research Institute, Babol University of Medical Sciences, babol, Iran; 4 Department of Healthcare Management, School of Public Health, Babol University of Medical Sciences, Babol, Iran

**Keywords:** Knowledge, Health belief model, Intention planned behavior, Subjective norm, Perceived control behavior, Preventive behavior, COVID-19 pandemic

## Abstract

**Background:**

This study aimed to investigate the integration of the health belief model (HBM) and the theory of intention to plan preventive behavior for COVID-19 during the pandemic.

**Methods:**

In a cross-sectional study, a sample of 480 adult participants from different outpatient clinics were recruited in the study*. T*he participant responded by self-report; the health belief model (HBM) scale, preventive behavior scale, subjective norms scale, the intention of planned behavior scale, and perceived control behavior scale were measured. The hypothesized causal path models were examined using SEM analysis.

**Results:**

The HBM had significant effects on perceived behavior control (β=0.60, P=0.001), the intended preventive behavior (β=0.32, P=0.001), and subjective norm (β=0.53, P=0.001). Subsequently, the intention of preventive behavior (β==0.39, P=0.001) and subjective norms (β=0.27, P=0.001) significantly affected the performance of preventive behaviors. The estimated fitting criteria showed that the hypothesized model fits relatively well.

**Conclusion:**

The health belief model with the integration of subjective norms, perceived control behavior and mediation by the intention of planned behavior in a pathway relationship explains well the preventive behavior of COVID-19. The findings present a deeper understanding of how integrating HBM and intended planned behavior enhances people’s preventive behavior against COVID-19.

Coronavirus diseae-19 (COVID-19) continued to spread in the different sub-groups of the population rapidly and it is a global public health emergency that results in a high cost for health resources and mortality (1). The corresponding psychological impact interfered with the health state of families in the general population, especially among healthcare workers and nurses (2-7). An updated data of world statistics indicated over 2 million deaths and about 165 million total cases and 143 million recovered/discharged worldwide the percentage of death from coronavirus accounted for about 2% of total cases (8).

 In the Islamic Republic of Iran, COVID-19 continues to spread over the population with health emergency condition and from 3 January 2020 to 16 February 2023, there have been 7,566,043 confirmed cases of COVID-19 with 144,789 death reported to WHO (9). Although, the mass vaccination and invention of a new therapeutic agent against COVID-19 are promising to prevent the disease in nearly future. The major strategy should emphasize preventive behaviors to control the spread of disease and thus decline the burden of disease globally and at the national level. 

Based on WHO guidelines preventive behaviors have been advised for people to stay at home as much as possible, frequently wash their hands, avoid gathering and to adhere social distance at least 1-2 meters away from each other, and avoid touching their faces to prevent or delay transmission of infection (10). However, some evidence shows emerging several increasing peaks in its incidence and mortality in the world and particularly in the Islamic Republic of Iran. This emerging increasing incidence in sequential periods indicates that adherence to the preventive command might have declined over time. Therefore, understanding and exploring the perceived belief of individuals that may be responsible for the preventive behavior of COVID-19 is of great interest from public health perspective against the covid-19 virus to spread.

Several theories of cognitive models of health belief have been developed (11-12). Of these, the health belief model (HBM) and the theory of intention to plan or cognitive planned theory have been recognized as determinants of health behaviors (12, 13). The HBM consists of several subscales of different constructs including perceived susceptibility, perceived seriousness, perceived barriers, perceived benefits, self-efficacy, and health motivation. In particular, it has been established that HBM influences the practice of women in breast cancer screening performance such as breast self-examination (12-15). On the other hand, the theory of planned behavior (TPB) assumes that the direct predictor of health behavior is the behavioral intention, which in turn is explained by health belief, subjective norms, and perceived behavioral control or self-efficacy i.e. perceived power to perform behaviors (11). The subjective norms correspond to the personal belief whether the reference groups support the behavior and their recommendation motivates to adhere to the individuals’ behaviors. However, the data are sparse regarding the effect of the integration of HBM and cognitive planned theory in the adoption of preventing behaviors of COVID-19 in the culture of the Iranian population. The framework hypothesized model of the integration effect of HBM and TPB on adopting preventive behaviors has been shown in Figure 1. Thus, the objective of this study was to adopt a preventive measure for COVID-19 and to develop a causal path model to explore the effect of integration of HBM components and cognitive panned theory in the preventive behaviors of COVID-19. More specifically, the specific objectives were as follows: 1) To adopt a preventive measure scale and HBM scale for COVID-19. 2) To explore the interrelation between components of HBM and their direct and indirect influence on preventive behaviors of COVID-19. 3) To examine the conceptual framework hypothesis of integration of HBM and cognitive panned theory with preventive behaviors of COVID -19. 4) To evaluate the fitness of the causal pathway model in the association of components of HBM, and the theory of planned behavior with preventive behaviors of COVID -19. Understanding this pattern helps the direction of future health plans and how to develop the education interventional strategy and action plan that focus on promoting health belief.

## Methods

**Study Design and Subjects:** This cross-sectional study was conducted with a sample of 480 adult individuals aged ≥18 years in the outpatient clinic of the affiliated hospitals and health centers of Babol University of Medical Sciences, North of Iran. A cluster sampling technique was performed and 8 clinics and health centers were selected within each clinic and health center, 60 participants (30 men and 30 women) were chosen consequently in the study. The allocated sample size of 480 enables to detection of an effect size of 0.15 in standardized coefficients of the causal path model with a 95% confidence level and 80% power. The subjects with a severe abnormal disability, recent CVA, severe dementia, and deaf-mute and those who did not consent to participate were excluded from the study. All participants were given written consent before enrolment in the study. The study protocol has been approved by the Ethical Board of the National Institute of Medical Research Development, Tehran, Iran (Ethic code: IR.NIMAD.REC.1400.106).

**Instruments and Data Collection:** The data were collected via a face-to-face interview with participants by trained nurses in outpatient clinics. The demographic data including age, sex, education level, and history of chronic diseases such as hypertension (HTN), diabetes, coronary artery, and cancer were collected at the first line. Also, the participants were asked whether they already had morbidity from COVID-19 and whether they were hospitalized. The data of the knowledge, preventive behavior, HBM, subjective norm, intended preventive behavior, and perceived control behavior were collected using the following scales.

**Knowledge of signs, symptoms scale:** The first scale measured the knowledge of signs, symptoms, transmission, and prevention of COVID-19 using a standard 15-item scale that was adopted by Zhong et al. (2020) (16). The correct response weighs 1 point and 0 for either an incorrect response or a lack of knowledge. Thus, the total score ranged from 0 to 15. We categorized this total score into three levels: 0-8 as low, 9-12: as moderate, and 13-15 as the high level of knowledge.

**Preventive behavior scale:** The second scale adopted the Covid-19 preventive behavior that measures the perceived likelihood of engaging in behaviors in reducing exposure to COVID-19. This scale includes the 19 items of commands of WHO guidelines including the five sub-scales of social distancing (SD), social isolation (SI), hygiene behavior (HB), healthy lifestyle (HL), and seeking information (SIN) of the COVID-19. 

The items of this scale compose of using a face mask when leaving home, washing hands regularly, avoiding public transportation, avoiding non-essential traveling, avoiding crowding places, avoiding shaking hands with others, keeping the social distance at 1-2 meters, avoiding hand contact on the surface of noise and eyes in outdoor, avoiding eating outdoors, avoidance of visiting other individuals with suspected of Covid-19, consumption of fresh fruit, vegetable and juice, doing regular exercise, and having sufficient sleep and seeking knowledge for the COVID-19 information. A 5-point Likert scale (1=not at all, 5= always) was used to measure the participants’ responses. All items had the same direction and a higher score indicates a greater of likelihood in preventive behaviors. The high validity and reliability of this scale have been reported in previous studies (17). In our data, the reliability of this scale as estimated by Cronbach’s alpha was 0.92.

**The Health Belief Model scale:** The third questionnaire was the HBM scale that has been implemented by Champion and it has been documented with good psychometric properties (12). We adapted this scale for coronavirus infection that composed of 37 items in seven different subscales including perceived susceptibility (5 items), perceived seriousness (7 items), perceived benefits (5 items) perceived barriers (5 items) perceived self-efficacy (5 items), perceived health motivation (6 items) and cues to action (5 items). The latter component as information cues to action composed of reading about the illness information, knowing about services, and consulting with others about once’ illness triggers self-care. The response rating scale of each item of this scale was ranged with a 5-point Likert scale (1=not at all, 5= always). Except for the subscale of barriers, in all other subscales, the higher score indicates the likelihood of more positive individuals’ beliefs. In the current study, the reliability coefficient was 0.87 as calculated by Cronbach’s alpha.

**Subjective nom scale:** The fourth scale measures the subjective norms that refer to the belief that an important person or group of people will approve and support a certain behavior as external pressure and also from internal person impression that a person feels personally obligates himself/herself to reduce the risk (11). This questionnaire is included a 6-item scale where 3 items are the recommendation from family, friends, and family physicians in engaging the preventive behaviors and the three other items are the personal subjective feeling as a personal obligation of his or her duties in risk reduction. The participants’ response rate of this scale is also on a 4-point Likert scale. The reliability coefficient of this scale was 0.79 in our analysis.

**Intended preventive behavior scale:** The fifth scale is a 4- item scale to measure the willingness and individuals’ intention of preventive behaviors. For example, “I am willing to implement the preventive behavior of coronavirus in my daily life: “I put a major effort to adopt the ways to counter the spread of coronavirus”; “I will do everything to reduce the impact of coronavirus”; “It is my responsibility to encourage my family, friend, neighbors, and others to adapt the way in engaging preventive behaviors”. The response rate of this scale also is on a 4-point Likert scale (1=strongly disagree to 4=strongly agree). Its reliability coefficient was calculated as o.85 in our data.

**Perceived behavioral control scale:** The seventh questionnaire measures perceived behavioral control which is defined as the perception of difficulty in acting a behavior (11). This scale measures the perception of one’s abilities and sense of control over the situation. It assesses the respondent’s self-efficacy and their perceived controllability of the behavior and it composes of a 4- item scale including “I am confident to manage the preventive behavior of the COVID-19, if I wish”, “For me, the adopting preventive behaviors of the COVID-19 is easy”, “The decision of adopting the preventive behavior is beyond my control” and “The adopting preventive behavior of the Covid-19 is entirely up to me”. The response rate is also a 4-point Likert scale (1=strongly disagree to 4=strongly agree) and its reliability coefficient was calculated as 0.77 by Cronbach's alpha in our data analysis.

**Statistical Analysis:** We applied the SPSS software of Version 24.0 and also the AMOS software of \Version 24.0 for data analysis. 

The descriptive statistics was presented by the median and interquartile range (IQR) for scored data and frequency and percentage for categorical data. In bivariate analysis, the Wilcoxon rank test and Kruskal Wallis test or nonparametric ANOVA model were used to compare different groups in preventive behaviors. The correlation of components of HBL, subjective norms, and intention /panned behavior with the preventive measure were examined by the Spearman correlation coefficients test. Additionally, the path analysis is performed to demonstrate the interrelationship of components of HBM, subjective norms, and intention to plan with Covid-19 preventive behaviors using structural equation modeling. The standardized direct and indirect coefficients are estimated and tested. The p-value less than 0.05 was considered a significant level. The fitness of hypothesized model with observed data was examined by SEM fitting criteria as recommended by Hu and Bentler (18) and Hooper et al. (19), including the goodness of fit index (GFI), adjusted goodness of fit index (AGFI), normed fitting index (NFI), root mean square error of approximation (RMSEA), comparative fitting index (CFI), incremental fitting index (IFI), parsimony goodness of fit index (PGFI), and Akaike information criterion (AIC).

## Results

**Descriptive statistics of demographic characteristics and comorbidity:** The mean age of participants was 39.5 (13.1) and ranged from 18 to 73 years. The characteristics of the study sample were shown in table 1.

 The majority of participants (53.6%) their age ranged from 18-39 years and only 8.1% were 60 years or older. The educational level of 86% at elementary or higher and only 5.8% were illiterate and roughly 67.1% were married. About 183 (38.1%) individuals had a history of COVID-19 morbidity. Out of 480 participants, only 18 (3.7%) were hospitalized due to COVID-19 morbidity. About 392 (81.7%) of subjects received the two-dose of COVID-19 vaccination and 39(8.1%) did not get it at all. The prevalence of chronic morbidities such as hypertension (HTN), diabetes, heart diseases (HD), and cancer were 17.9%, 10.4%, 11.7%, and 2.3% respectively. Roughly, 17.3% of the study samples were current smokers, 4.2% were ex-smoker and the remainder (75.5%) had no history of smoking at all. The majority of participants (40.8%) had a moderate level of knowledge regarding signs, symptoms, and prevention of COVID-19, 37.1% high level of knowledge, and the remainder (22.1%) had a low level. 

**Gender difference in HBM and preventive behavior:** Table 2 shows the mean (SD) of scores in health belief model sub-scales and the average of total scores of different scales used in the study according to gender. There were no statistically significance in the mean of total scores of knowledge, preventive behavior scale, and HBM scale between genders except for susceptibility, and a clue to action that men had higher average scores than women (P=0.004 and P=0.001, respectively). However, women had a slightly higher level of knowledge of COVID-19 and a slightly higher level of education than men but non-significantly. Overall, the observed average scores of all scales studied were almost higher than the average of the corresponding scales. The participants of both genders reported lower barriers and higher benefits, higher health motivation, and self-efficacy about preventive behavior in the HBM scales. 

**Table 1:** Demographic and Clinical characteristics of study population

**Characteristics**	**Categories**	**n (%)**
**Age group**	18-39(y) 40-59 ≥ 60	257(53.6) 184(38.3) 39(8.1)
**Sex**	Male Female	240(50.0) 240(50.0)
**Education**	Illiterate Primary Elementary/high school University level	28(5.8) 36(7.5) 189(39.4) 227(47.3)
**Marital Status**	Single Married Divorced Widow	133(27.7) 322(67.1) 13(2.7) 12(2.5)
**Smoking**	Not at all Ex-smoker Current smoker	377(78.5) 20(4.2) 83(17.3)
**HTN**	No Yes	394(82.1) 86(17.9)
**Diabetes**	No Yes	430(89.6) 50(10.4)
**Heart disease**	No Yes	424(88.3) 56(11.7)
**Cancer**	No Yes	469(97.7) 11(2.3)
**COVID-19**	No Yes	297(61.9) 183(38.1)
**Hospitalization due to COVID-19**	No Yes	362(96.3) 18(3.7)
**Vaccination**	Not at all 1^st^ dose 2nd dose	39(8.2) 48(10.0) 392(81.8)

HTN: Hypertension; COVID-19: Coronavirus disease-19

**Table 2:** The Mean ± SD of different scales of COVID-19 according to gender

**Scales**	**Male** **(n=240)** **Mean ± SD**	**Female** **(n=240)** **Mean ± SD**	**P-value**
**Knowledge**	10.83 ± 3.01	11.07 ± 2.88	0.39
**Susceptibility**	2.29 ± 0.91	2.07 ± 0.74	0.004
**Seriousness**	1.93 ± 0.75	2.05 ± 0.72	0.07
**Benefits**	3.78 ± 0.94	3.77 ± 0.86	0.83
**Barriers**	1.61 ± 0.72	1.57 ± 6.65	0.47
**Confidence/Self-efficacy**	3.45 ± 0.89	3.21 ± 0.83	0.92
**Health motivation**	3.21 ± 0.83	3.18 ± 0.83	0.07
**Clue to action**	2.80 ± 0.87	2.43 ± 0.83	0.001
**Total score of HBM**	114.58 ± 17.14	112.01 ± 15.45	0.10
**Total score of preventive behavior **	62.90 ± 15.29	62.93 ± 13.03	0.98
**Total score of subjective norms**	20.13 ± 3.19	20.18 ± 2.82	0.87
**Total score of intention of preventive behaviors **	13.89 ± 2.31	13.83 ± 2.22	0.72
**Perceived behavioral control **	12.45 ± 2.06	12.34 ± 1.88	0.52
Scale range of total score for knowledge: 0 - 15 Scale range of total score for HBM: 37 – 185 Scale range of total score for preventive behaviors: 19 -95 Scale range of total score for subjective norms: 6 – 24 Scale range of total score for behavioral intention: 4 - 16 Scale range of total score for Perceived control behaviors: 4 – 16			

**Correlation structure between the total scores of different scales:** Table 3 shows the Spearman correlation between the total scores of the different scales studied. A significant positive correlation was observed between knowledge, and health belief model scores with preventive behavior of COVID-19 (r=0.36 (P=0.001), r=0.65 (P=0.001), respectively). Also, the score of subjective norms, preventive behavior intention, and perceived control behavior has been positively associated with preventive behavior of COVID-19 (r=0.57(p=0.001), r=0.61 (P-=0.001) and r=0.39 (P=0.001), respectively).

**Figure 1:**
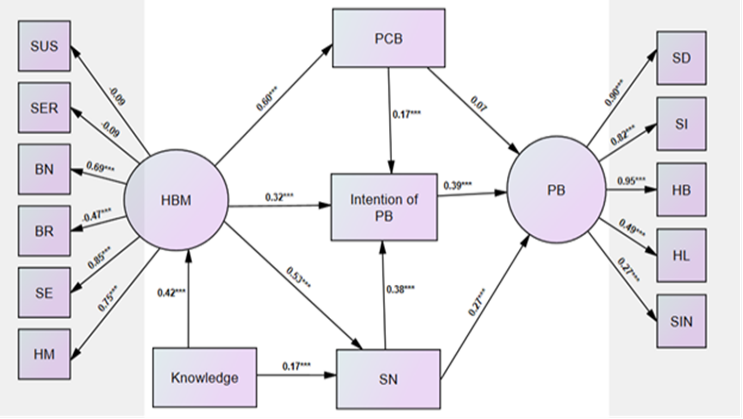
The estimated standardized regression coefficients and loading factors in the hypothesized causal path model using SEM analysis

**The causal path effect in hypothesized model and summary statistics of fitting criteria:** Figure 1 shows the standardized loading coefficients and path effects of the structural equation model in the explanation of the practice of preventive behavior of COVID-19. Health belief had significant effects on perceived behavior control (β=0.60,) P=0.001), the intention of preventive behavior (β=0.32, P=0.001), and subjective norm (β=0.53, P=0.001) and subsequently, the intention of preventive behavior (β==0.39, P=0.001) and subjective norms (β=0.27, P=0.001) had a significant effect on the performance of preventive behaviors. The loading coefficients of all 5 subscales of preventive behavior were significant (P=0.001) while two subscales of susceptibility and seriousness did not appear to be significant but the loading coefficients of the other four dimensions including benefits, barriers, self-efficacy, and health motivation were statistically significant in the HBM (P=0.001), and the higher score of barriers harms the HBM model. Table 4 summarized the fitting criteria in the examination of hypothesized model. The estimated fitting criteria showed that the hypothesized model fits relatively well with our data and the fitted model well explains the preventive behavior of COVID-19. 

**Table 3:** Spearman correlation (p-value) between total scores of different scales for evaluation preventive behavior of COVID-19

**Scales**	**1**	**2**	**3**	**4**	**5**	**6**
**Knowledge**	1(-)	0.37 (0.001)	0.36 (0.001)	0.38 (0.001)	0.37 (0.001)	0.31 (0.001)
**HBM**		1(-)	0.65 (0.001)	0.54 (0.001)	0.56 (0.001)	0.41 (0.001)
**PB**			1(-)	0.57 (0.001)	0.61 (0.001)	0.39 (0.001)
**SN**				1(-)	0.61 (0.001)	0.41 (0.001)
**BI**					1(-)	0.52 (0.001)
**BC**						1(-)

HBN: Health belief model; PB: Preventive behavior. SN: Social Support and Subjective norms, BI: Behavioral intention, BC: Behavioral control

**Table 4:** Model summary of fitting criteria

**NFI**	**RFI**	**IFI**	**TLI**	**CFI**	**RMSEA**	**CMIN/df**
0.87	0.81	0.89	0.79	0.85	0.09	5.29

NFI: Normed fitting index, RFI: Relative fitting index, IFI: Incremental fitting index TLI: Tucker-Lewis index, CFI: Comparative fitting index, RMSEA: Root mean square error of approximation, CMLN: Chi-square, df: degree of freedom

(*** P=0.001, HBM: Health belief model, SUS: susceptibility, SER: Seriousness, BN: Benefits, BR: Barriers, SE: Self-efficacy, HM, Health motivation, PCR: Perceived control behavior, PB, Preventive behavior, SN: Subjective norm, SD: Social distance, SI: Social isolation, HB: Hygiene behavior, HL Healthy lifestyle, SIN: Seeking information)

## Discussion

This study was planned to develop a causal path model to determine the role of an integrated model of HBM and the theory of intention to plan the adaptation of preventive behavior of COVID-19 and to estimate the standardized effect of components of this integrated model on preventive behavior. In particular, the preventive role of perceived susceptibility, seriousness self-efficacy, perceived benefits, perceived barriers, and health motivation were quantified for HBM and the mediating role of the intention of planned behavior has been shown in adapting preventive behaviors of COVID-19. The results of our unifying causal path model provide an action plan as a guideline in adapting preventive behavior for health decision-makers as a coping strategy for the management of control of the spread of the COVID-19 epidemic. This novel knowledge provides an innovation strategy through the establishment of the components of the theory of planned behavior and HBM in health interventional programs for promoting preventive measures in future health management strategies in the population.

In the current study, we found the integration of the health belief model and planned intended theory explains the preventive behaviors of COVID-19 by the path causal relationship. We did not find a significant difference between gender in the health belief model and preventive behavior scales but women had a slightly higher level of knowledge of COVID-19 and also education level than men non-significantly. In contrast, a cross-sectional study by Shahnaz et al. (2020) assessing preventive health behavior from COVID-19 in the North of Iran showed that the mean score of preventive behaviors was higher in females than males (20). 

Meanwhile, in their study, self-efficacy and perceived benefits have been positively associated with the score of preventive behaviors while the score of perceived barriers decreased the score of preventive measures for COVID -19 which were rather similar to our findings. However, their analysis was limited to multiple linear regression and they did not explore the causal model to examine the causal pathway of components of HBM on preventive behaviors. Similarly, Hungarian women were more likely to engage in preventive behavior against COVID-19 and they perceived a greater risk of disease also disease information was more frequent in women than men (21). Another study reported the application of HBM with preventive behaviors of COVID-19 among adolescents in the central part of Iran (22) showed a significant correlation between the adolescents’ preventive behaviors and their self-efficacy, perceived benefit, and perceived severity while there was a significant negative association between adolescents’ predictive behaviors and perceived barriers. An internet-based study in the North of Iran indicated that a positive significant association between preventive behaviors and perceived susceptibility, perceived benefits, and perceived barriers have been reported. Overall, HBM explained 26% of the variance of preventive behaviors of COVID-19, among which perceived self-efficacy was the most potent predictor (23). While in our findings, the influence of components of HBM on preventive behavior through the mediating of the intention of planned behavior has been explored. This integrated effect has a greater impact on the preventive behavior of COVID-19.

Several factors can explain the adoption of preventive behavior in health practice as our hypothesized model showed. Similarly, a cross-cultural study in China and Israel showed a 3-component model: confidence, complacency, and constraint are predictors of adopting specific behaviors (vaccination) (24). Their findings suggested this three-component model can be generalized from getting a vaccination to adopting avoidance behaviors that can be used in cross-culture. In another report of adaptation preventive behaviors in Singapore of 897 adults in a web-based survey, the participants who perceived higher COVID-19 risk had a higher self-efficacy and adaptation of preventive measures. Self-efficacy was a stronger predictor of behavior changes (25). Robert et al. investigated the predictors of COVID-19 preventive behaviors using a developed sequential mediating model among US college students (26). Their conceptual model of sequential path included fear of COVID-19, information receptivity, perceived knowledge, and self-efficacy and followed by preventive behaviors. This sequential mediation path model showed that the fear of COVID-19 leads individuals to seek out more information regarding COVID-19, which increases their perceived knowledge and fosters self-efficacy that influences preventive behaviors (26).

Our findings showed that subjective norm is a stronger predictor of both intentioned planned and adopting behavior followed by knowledge level. Similar findings have been demonstrated in the study of Chilean students (27). In addition, based on protection of motivation theory reported that the treatment and coping appraisal, and intention were the predictors of preventive behavior (28).

In the current study, our findings showed that over three-quarters of our study population had adequate knowledge of the signs, symptoms, and prevention of COVID-19. While the majority of Turkish adults had inadequate knowledge of COVID-19 but highly engaged the preventive behaviors (29). The higher knowledge of our study population may be explained by the higher level of educational level that over one-third of our study samples had education at the university level.

The extension of integration of HBM and TPB is a matter of interest in adopting healthy behaviors. In the state of the epidemiologic transition of COVID-19, the most important is to adopt preventive behaviors in cross-cultures. Since the HBM is influenced by the cultural condition, regarding the control of the spread of COVID-19, the data was already sparse in the Iranian culture to explain the adoption of preventive behaviors through the causal pathway association by an action plan that integrates the HBM and TPB in the future interventional program.

The cross-sectional nature of this study might have limited in the exploration of the causal association and also the direction of the path association. Thus the interpretation of results must be cautious. The other limitation is that the data were obtained from the self-reported questionnaires that may not be verified and adjusted by clinical exam. This self-reported data may be either exaggerated or underreported the findings. However, such misclassification is non-differential to affecting factors of preventive behavior. Moreover, future longitudinal studies may compensate for the direction of the path to be established in the causal path model.

The health belief model with the integration of subjective norm, perceived control behavior and mediation by intention-planned behavior in a path-way relationship explain the preventive behavior of COVID-19. Based on the findings of this research, an action plan might incorporate the perceived risk and perceived self-efficacy in the integration cognitive model of intention to plan in future work of the interventional health education study for the population to examine the promotion of adherence to preventive behaviors. Further studies can be performed in more restricted and controlled conditions of a randomized clinical trial to explore what extent and how the related cognitive models are effective in adapting preventive behaviors in epidemic conditions.
